# Epidemiological Characteristics of the African Swine Fever Genotype II Epidemic in Domestic Pigs in Lombardy (Northern Italy) in 2023 and 2024

**DOI:** 10.3390/v17030327

**Published:** 2025-02-26

**Authors:** Silvia Bellini, Sara Salvato, Martina Bernardis, Mario Chiari, Federico Martinello, Luigi Galimberti, Valentina Maggiano, Francesco Feliziani, Marco Farioli, Giovanni Loris Alborali

**Affiliations:** 1 Istituto Zooprofilattico Sperimentale della Lombardia e dell’Emilia Romagna (IZSLER), 25124 Brescia, Italyvalentina.maggiano@izsler.it (V.M.);; 2 Direzione Generale Welfare, Regione Lombardia, 20124 Milan, Italy; 3 Agenzie di Tutela della Salute (ATS), 27100 Pavia, Italy; 4 Agenzia di Tutela della Salute (ATS), Città Metropolitana di Milano—Distretto Veterinario Alto Lodigiano, Sant’Angelo Lodigiano, 26900 Lodi, Italy; 5 Laboratorio Nazionale Referenza Pesti Suine (PSC—PSA), Istituto Zooprofilattico Sperimentale dell’Umbria e delle Marche (IZSUM), 06126 Perugia, Italy; 6 Agenzie di Tutela della Salute (ATS), Insubria, 21100 Varese, Italy

**Keywords:** African swine fever virus, domestic pigs dynamics, risk factors, outbreak response, disease control

## Abstract

African swine fever (ASF) is a severe hemorrhagic disease of suids caused by the African swine fever virus (ASFV). In 2023, the introduction of genotype II ASFV into Lombardy was a cause for serious concern; the region is home to approximately 50% of the national pig population and is of economic importance to the processing industry of the entire country. Since then, two ASF epidemics have resulted in a total of 30 outbreaks in domestic pigs in the same areas of Lombardy, where the disease is endemic in wild boars. The results of the control activities conducted in the affected areas seem to indicate the establishment of self-sustaining infection cycles in the wild boar population with spillover and spillback events occurring between wild boars and domestic pigs. This manuscript describes some epidemiological features of the ASF epidemics in Lombardy with the aim of providing useful information to combat the disease.

## 1. Introduction

African swine fever (ASF) is a highly lethal viral disease of domestic pigs and Eurasian wild boar. It is caused by African swine fever virus (ASFV), a large DNA virus of the *Asfarviridae* family, of which at least 24 several viral genotypes are recognized. ASFV is highly resistant in meat products and it has been found to sustain infectivity for long periods in different matrices [1].

In 2007, genotype II of the ASFV was transported from Southeast Africa to Georgia [2] and has subsequently spread to Russia, Europe, China, Southeast Asia, and Central America, with devastating socioeconomic consequences. In the European Union (EU), ASFV was introduced in 2014 and has since moved westward, affecting several EU countries where the disease has become endemic in wild boar, with some spillover to domestic pigs. The current wide spread of the disease and the endemicity of ASFV in the field make eradication of the disease increasingly difficult [3].

In January 2022, the first occurrence of genotype II ASFV was reported in wild boar in a rather impervious and forested area in the northwestern part of Italy [4]. Given the characteristics of the affected area, it was immediately clear that it would not be easy to implement adequate control measures to prevent the spread of infection from this area. In fact, ASF gradually spread in wild boar, and on 21 June 2023, a wild boar was found positive for ASFV in the municipality of Torretta di Bagnaria (Lombardy region, Pavia Province), on the border with Piedmont. Two months later, ASFV was detected in a pig farm (outbreak) in an area where the disease was present in wild boar. Afterward, in 2023 and 2024, Pavia Province was affected by two ASF epidemics.

In 2023, nine outbreaks of ASF were detected and eradicated just over a month after the epidemic began. Nevertheless, the infection continued to spread among wild boar up to the Ticino Park, a 91,410 hectare (22,588 acre) natural green area located along the banks of the Ticino River.

At the end of July 2024, ASFV was reintroduced into domestic pigs, with the first outbreak identified in the Ticino Park, where the infection probably spread from wild boar to domestic pigs. Subsequently, another 20 outbreaks were identified in pig farms in the surrounding area, with transmission following typical between-farm transmission routes.

**Figure 1:**
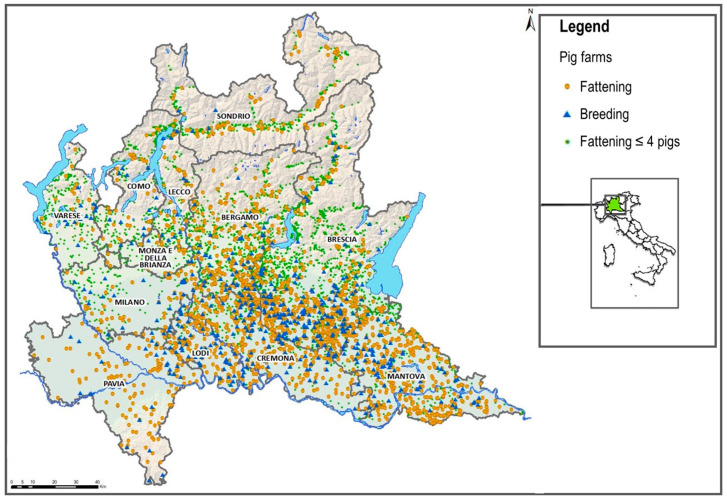
Distribution of pig farms in Lombardy.

The presence of ASFV in northern Italy was immediately a cause for serious concern. Indeed, Lombardy is an area of intensive pig production, home to about 50% of the national pig population, and the Lombard pig sector is also of economic importance for the processing industry, which aims to produce high-quality pork products. The provinces of Brescia, Mantua, and Cremona of Lombardy (Figure 1) are densely populated areas for livestock (pigs, cattle, poultry) and the most suitable areas for pig farming.

Conversely, agriculture is less intensive in the province of Pavia, and often different agricultural activities coexist on the same farm, such as pig farming, wine, rice or other grain production.

As soon as ASFV was detected in domestic pigs in 2023, surveillance and control activities were implemented to stop the spread of the infection [5]. To enhance early detection, in the affected area, all dead pigs, regardless of clinical signs, had to be tested to exclude ASF as the cause of death. The epidemiological investigations and control measures adopted were all in accordance with national and European regulations (Regulation (EU) 2020/687) [6]. In order to understand the real extent of the epidemic and to improve the early detection of the disease, some provisions were also immediately adopted in the rest of the regional territory, and rendering companies were asked to immediately report any increase in the frequency of carcass disposal from pig farms to the veterinary services. In one-and-a-half months, more than 1500 farms were checked, covering about 75% of the intensive pig herds of the region and it was confirmed that ASFV was restricted to the province of Pavia [5].

The epidemiological investigations were performed with particular attention to identify the possible source of infection, carry out rapid tracing, and break the cycle of ASFV transmission.

Previous studies by EFSA reported that the specific routes of ASFV introduction into pig farms could only be identified in very few of the outbreaks for which detailed investigations were carried out. Inadequate biosecurity was likely to have contributed to the introduction of ASFV into domestic pig farms through indirect contact via contaminated fomites or the environment [7]. In addition, it was noted that ASF outbreaks in pigs were correlated in time and space with cases in wild boar, suggesting a link between the risk of introduction and the level of environmental contamination. However, in these cases, direct contact with infected domestic pigs or wild boar could be excluded as a probable route of introduction of the ASFV [7].

The aim of this manuscript is to present some epidemiological features of the ASF epidemic in domestic pigs in Lombardy, with an analysis of the main risk factors identified in the outbreaks during the epidemiological investigations. Finally, some conclusions and lessons learned from this epidemic are reported.

## 2. Materials and Methods

Five sources of information were used for this study:

1. Epidemiological investigation of the outbreaks;
2. The Istituto Zooprofilattico Sperimentale della Lombardia ed Emilia Romagna (IZSLER) Information System collecting the results of the diagnostic tests;
3. The National Animal Health Information System (SIMAN), which contains the complete list of ASF cases in domestic pigs and wild boar;
4. The national database (BDN) containing the list of pig farms operating in the country with details of property, type of farming, animals reared in the establishment, and geographical coordinates;
5. Veterinary information systems of the Central Veterinary Authority (VETINFO), which collect and manage health and non-health data aimed at the governance of the national Animal Health and Food Safety control programs, including the registration of animal movement and farm biosecurity. Access to the system requires authentication.

Data on pig movements and data referring to the characteristics of pig holdings were extracted from the BDN, which is the national database accessible via VETINFO. In the BDN, domestic pigs are categorized according to the pig numbers, with farms raising less than five pigs considered non-commercial farms (pigs are kept for family consumption) and farms with five or more pigs considered commercial farms. This database also contains details on the production orientation of the farm (fattening, breeding) and on the type of breeding (indoor, outdoor).

Data extracted from the SIMAN and BDN databases were mapped using QGis software (version 3.34) [8]. The statistical analysis was performed using the software R version 4.4.2 [9]. The spatial distribution of the outbreaks was assessed using kernel density estimation (KDE), a non-parametric method to estimate the probability density function of a random variable based on kernels as weights. The kernel radius was set to 1 km.

All outbreaks of ASF were investigated according to Commission Delegated Regulation (EU) 2020/687 [6], which lays down the rules for the prevention and control of certain listed diseases, and the epidemiological investigation was performed in accordance with the format established by the Central Veterinary Authority for the collection of animal registration details, anamnestic and clinical information, risk factors and other information useful for the tracing of other outbreaks. This form included farm details (location and farm owner), animal data (population size, production type), farm network characteristics (number and type of relationships with other farms), animal movements, external visits to the farm (veterinarians, breeders, salesmen), and clinical evaluation (number of dead and symptomatic pigs, date of disease suspicion, date of first symptom identification, type of symptoms, location of positive animals.

The information collected during the epidemiological investigation was supplemented by the results of the diagnostic activities carried out on the farm, which included the number of pigs serologically and virologically tested; i.e., the number of pigs detected as ASFV-positive and ASFV-antibody-positive, respectively. ASF diagnosis was performed as described in the World Organization for Animal Health Manual [10]. For tracing purposes, the risk period was considered to be twice the maximum ASF incubation period (fifteen days) (15 × 2 = 30 days). The traced farms were placed under restriction by the Competent Veterinary Authority, with a ban on animal movements and regular weekly clinical visits to assess the health status of the animals and sampling of animal tissues from sick and dead animals for real-time PCR analysis.

The epidemiological investigations were conducted upon confirmation of the outbreak by veterinarians of the Local and Regional Veterinary Services and staff of the Epidemiological Unit of the IZSLER. The form is available on the Ministry of Health website, https://www.vetinfo.it/ (accessed on 20 December 2024), where the completed epidemiological investigation is also uploaded. The results of biosecurity checks are also recorded in the same database.

In accordance with EU legislation, all outbreaks were eradicated by culling, and the carcasses were safely disposed of by rendering.

## 3. Results

**Figure 2:**
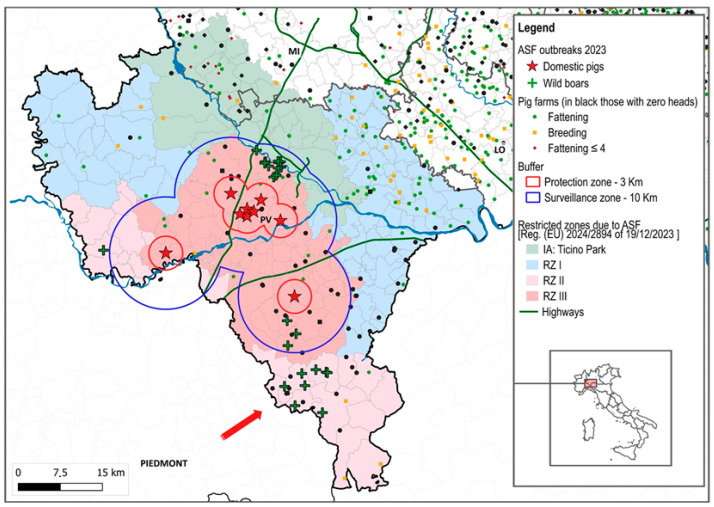
ASF outbreaks in Pavia province in 2023.

**Figure 3:**
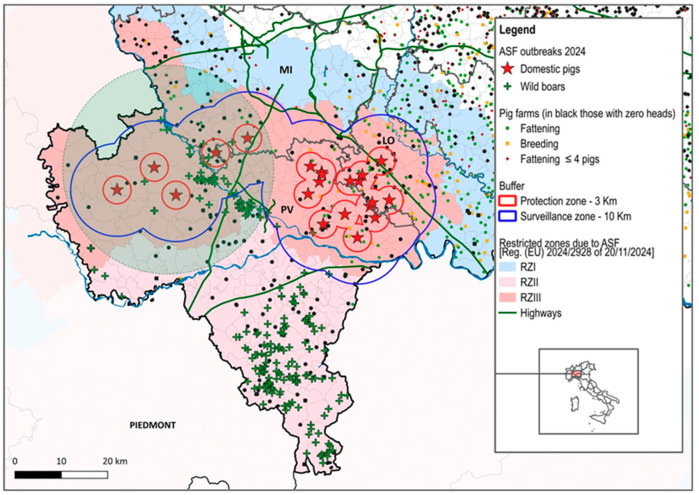
ASF outbreaks in 2024—the area of the province highlighted in green was also affected by ASF in 2023.

In the period 2023–2024, Lombardy was affected by two ASF epidemics in domestic pigs. The first started on 25 August and ended on 27 September 2023 with nine outbreaks, while the first outbreak of the second epidemic was detected on 25 July and ended on 18 October 2024 with 21 outbreaks (Figure 2 and Figure 3). The attack rate in Pavia was 5% in 2023 and 7.3% in 2024. The provinces of Milan and Lodi were involved in the epidemic in 2024, with an attack rate of 2.4% and 3.7%, respectively (Table 1).

In 2023, the majority of outbreaks occurred in fattening farms (8/9), while in 2024, 10 outbreaks (10/21) were detected in breeding farms, and the rest of the outbreaks were in fattening farms. Therefore, in the period 2023–2024, a total of 30 outbreaks were detected in Lombardy; of these, 19 were in breeding farms and 11 in fattening farms. It is worth remembering that at the time of the first outbreak in the province of Pavia, pigs from non-commercial and outdoor farms had already been slaughtered in the affected area, in order to avoid the spread of ASFV from feral to domestic pigs.

**Figure 4:**
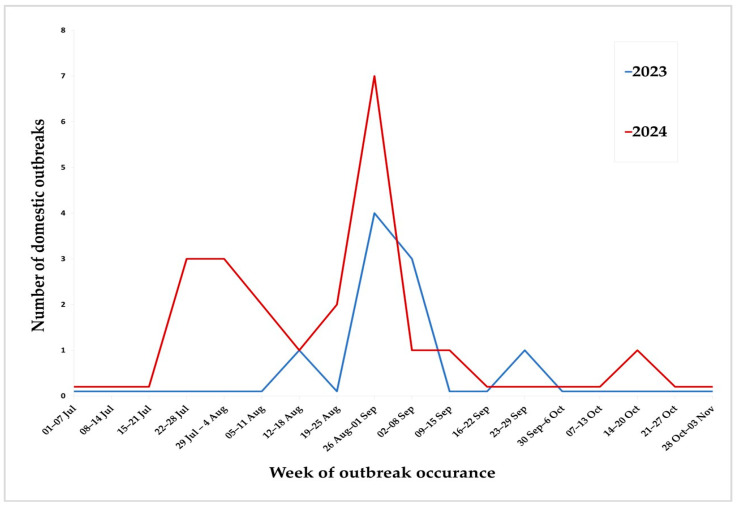
Epidemiological trend of 2023–2024 ASF epidemics.

The average number of animals in breeding farms was 4336 (min = 122, median = 3080, max = 12,548), while in fattening farms it was 2805 (min = 4, median = 1335, max = 19,615). In both periods, the epidemic peak was recorded at the end of August (Figure 4).

In the 2024 epidemic, only two outbreaks were detected due to clinical symptoms (1 and 4, Table S1); the others were identified through enhanced passive surveillance. In fact, all dead pigs in the affected area, regardless of symptoms encountered, were tested to exclude ASF.

The average prevalence (number of cases out of the total animals present on the farm) in the 2024 outbreaks was 3.64% (min = 0.01%, median 0.23%, max 52.46%). We excluded outbreaks in which epidemiological investigation occurred concurrently with the culling of the animals (stamping-out) from the prevalence calculation. Indeed, in such circumstances it was difficult to check the real health status of the animals.

The first ASF outbreaks of the two epidemics had similar characteristics. The epidemiological investigation suggested potential ASFV introduction via indirect contact with wild boar (human factor) and neither outbreak generated secondary outbreaks.

In 2023, the first outbreak occurred in a medium-small fattening farm (166 pigs) where, in addition to pig farming, various agricultural activities in the surrounding areas were carried out by the owner. It is most likely that ASFV was inadvertently introduced to the farm with vehicles or with the fodder produced on farm fields, which was also used to feed the pigs and as bedding (straw). In 2024, the first outbreak was detected in a breeding farm located in a municipality of the Ticino Park, where several cases of ASF had been reported in wild boars near the farm. On this farm, the animals were well cared for, but due to their age, the owners had difficulties in maintaining an intact biosecurity system, although they had implemented all the biosecurity law requirements [11].

In 2023, a central role in the spread of the infection was played by the second outbreak, which occurred in the municipality of Zinasco and led, directly or indirectly, to the following outbreaks; the case has already been described in a previous publication [5], and the results of the tracing activities are reported in Table S1. It is still important to remember that one of these outbreaks occurred in an “animal sanctuary” near the Ticino Park, where pigs were kept for recreational purposes. In this outbreak, despite the small number of animals, it was very difficult to carry out control and eradication activities, which were delayed by the involvement of the media and activist groups, who resorted to obstructionist tactics against the authorities to avoid the culling of animals. The delay in eradicating this outbreak and the interaction with an infected facility by groups of citizens not used to handling animals, especially in an emergency situation, may have led to the spread of ASFV to wild boar in the Ticino Park, an area that was not previously affected by the disease and from which the infection returned to domestic pigs in July 2024, thus causing the second ASF epidemic season in domestic pigs.

In 2024, it was a farm in Vernate, a municipality on the edge of the Ticino Park, that started the cycle of ASFV transmission between pig farms, also involving the supply chain. The owner of this farm had also worked in the loading and unloading of animals from farms in the supply chain, from where the infection was then transmitted between farms in the supply chain. In 2024, the peak of the epidemic was reached at the end of August, when ASF affected a few farms in the province of Lodi, on the border with Pavia. One of these outbreaks occurred in a breeding farm that shared personnel and means of transport with the associated fattening farms located close to the farm itself. Two outbreaks were reported in September, but by the time they were identified as infected, the herds had already been seized. This was because they had been traced back to outbreaks detected at the end of August (same personnel, same means of transport) and had already been identified for preventive culling.

In 2024, the last outbreak occurred almost a month after the previous one. For this outbreak, the origin remained uncertain. What is certain, however, is that the infection began and remained in the most external facility of the farm, where renovation work had begun with external workers at times compatible with the incubation period of the disease. Some entrances to the external facility had been opened to remove building materials stored inside the farm during the summer. The first positive pigs were identified near one of these entrances and the infection remained localized in the adjacent boxes; in this case, we assumed that the probable introduction of the infection could be the consequence of indirect contact with contaminated material or due to the disposal of feed given to the pigs by the external workers. In our records, this outbreak remains classified as an outbreak of unknown origin. Nevertheless, it is worth mentioning that in three outbreaks identified in 2024, there were renovation works carried out on the farm and the first evidence of the infection was near the works in progress.

Forty percent of the outbreaks (12/30) occurred in the protection zone of other outbreaks. In 2023, apart from the first outbreak, which did not generate a secondary outbreak, most of the outbreaks occurred in an area of approximately 3 km around the first outbreak in Zinasco. Those beyond 3 km were epidemiologically linked to the outbreaks within the 3 km area [5].

**Figure 5:**
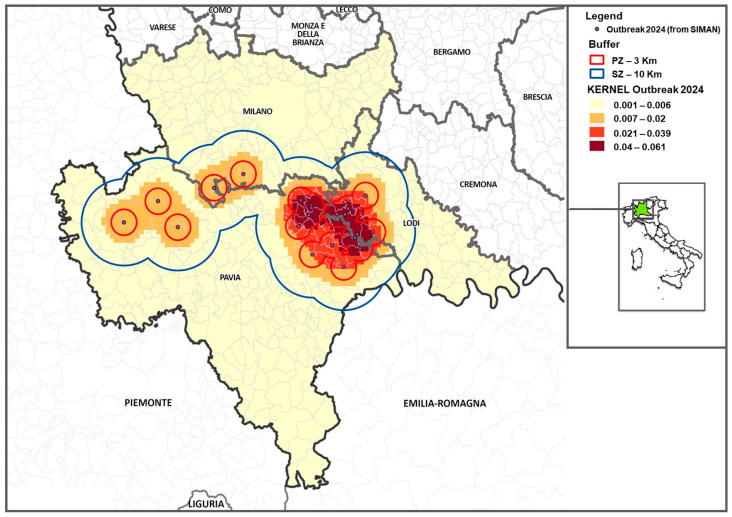
Kernel representation of ASF outbreaks detected in Lombardy in 2024.

In 2024, the first five outbreaks occurred in an area affected by ASF in 2023, where the population density in 2024 apparently was not sufficient to support proximity transmission, as the farms affected in 2023 had not been repopulated and therefore the pig density was low; in the surveillance zones of the first five outbreaks, the median number of pigs per km^2^ was 21. Aggregation of ASF outbreaks could be identified in the second part of the 2024 epidemic, when the disease affected a new area, on the border between Pavia and Lodi, where the density of pigs was 268.2 pigs per km^2^. In Table S1, the kernel values of the outbreaks that occurred in 2024 are reported, whereas the outcome of the elaboration is shown in Figure 5.

In 2023, 127 farms were traced due to contacts with infected farms during the risk period, while in 2024, 543 farms were traced. All these farms were placed under restriction and inspected by the veterinary services. Vehicle movement (load of pigs, feed, whey, dead pigs) was the most frequently traced route of transmission, accounting for 77.2% of tracings in 2024.

## 4. Discussion

In most of the ASF outbreaks that have occurred in Lombardy, the possible sources of infection were identified, which is essential to stop the infection from spreading. However, this is only possible if investigations are carried out promptly, accurate records of farm movements are kept, and farmers cooperate.

The enhancement of passive surveillance was very helpful in anticipating ASF diagnosis; indeed, half of the outbreaks in 2024 were detected before the identification of clinical signs and when mortality was still below the normal farm threshold. This aspect is very relevant for the purposes of disease control given that it shortens the farm high-risk period, reduces virus production, and avoids further spread of the infection.

The results of the investigations showed that during the period of risk, some outbreaks were exposed to several possible routes of introduction of the infection, all listed in Table S1, and it was not always possible to identify which one was effective in transmitting the infection.

Movement of infected animals is normally very effective in transmitting the infection [12], whereas the epidemics described in this study occurred only twice, in 2023. This can be explained by the fact that in 2023 most of the outbreaks occurred in fattening farms and the pigs were not at the end of the fattening cycle at the time of the epidemic; therefore, no animal movement occurred in most of the outbreaks. In contrast, in 2024, the majority of the outbreaks occurred in areas that had already been regionalized due to the presence of ASFV in wild boar; therefore, pig movements were already regulated. In 2023, two infected farms were involved in the movement of potentially infected animals, only one of which led to a secondary outbreak on a small farm in the municipality of Dorgali in Sardinia [13].

As reported in a recent study conducted by EFSA [14], during the ASF epidemic in Lombardy, there was also a spatial and temporal correlation between the presence of the disease in wild boar and domestic pigs. In both epidemic periods, wild boar may have played a role in the initial introduction of ASFV into domestic pigs, while in subsequent outbreaks the spread occurred through typical farm-to-farm transmission routes. To date, in some areas of the provinces of Pavia and Lodi, affected by ASF in domestic pigs, the disease has never been identified in wild boar. Therefore, given the experience of the Ticino Park, it is also necessary to adopt measures aimed at avoiding the transmission of the infection from pigs to wild boars, in order to prevent the establishment of ASFV transmission cycles between domestic pigs and wild animals, which may also lead to the expansion of the infection front.

The epidemiological investigations carried out in the infected farms made it possible to trace the movements of people (workers, veterinarians, technicians) and vehicles at risk, such as trucks transporting feed, animals, and carcasses. These contacts are highly concentrated among farms that are part of a supply chain, which often also share technicians and veterinarians.

Approximately 50% of the outbreaks, 70% in the second epidemic period, occurred in farms that were part of supply chains, which normally means having a common source of piglets and genetic material, the same feed supply, sharing vehicles, slaughterhouses, technical personnel, and veterinarians. The factors mentioned, in the event of the introduction of a pathogen, can facilitate the transmission of the infection and this is true regardless of the disease and the animal species involved [15]. Nowadays in both the pig and poultry sectors, a large proportion of commercial farms are organized in a supply chain; this farming method makes it possible to maintain uniform production standards, contain costs and control the market. However, in the farms of a supply chain, the exchanges between the various components of the system itself (farms, vehicles, raw materials, technical personnel, veterinarians, slaughterhouses, plants) are very intense and this allows the rapid spread of diseases in the event of the introduction of infection.

Forty percent of the outbreaks occurred in the protection zone of previous outbreaks. Indeed, transmission of infection between neighboring herds is a fairly common mode of transmission for epidemic diseases, especially when they occur in densely populated areas. Proximity between farms is known to be a risk factor in epidemiological analysis and control of livestock epidemic diseases, even in the absence of accurate information on disease spread between farms [12,16,17,18]. This seems to be in line with what was recently published by EFSA, where it was reported that the minimum distance to outbreaks of ASF in domestic pigs was significant in the regression model they used. Case farms had a median distance to the nearest outbreak of 3.8 km, which is in line with the findings of Boklund et al. (2020) [19] and the EFSA report in 2023 [20], which analyzed the evolution of ASF outbreaks in 2022 [14].

Considering the characteristics of spread of the disease, it was decided to analyze the 2024 outbreaks also as a function of the density of pigs in the affected areas, given that it is known that the density of susceptible animal species is one of the elements influencing the spread of a disease [12,16,21]. The results indicate that the density of outbreaks was higher in territories where the density of pigs was higher.

It is still important to remember that in 2023, the delay in eradicating the outbreak in the “sanctuary”, due to the protests promoted by activists, may have led to the spread of the ASFV to wild boars in the Ticino Park, an area that was not previously affected by the disease and from which the infection returned to domestic pigs in July 2024, thus causing the second epidemic season of ASF in domestic pigs.

Finally, it may be useful to mention that since the outbreaks occurred during the summer, in some of these farms the animal care staff was on vacation and replaced by temporary staff who most likely did not have the same level of competence as the regular staff, another factor that may have contributed to weakening the biosecurity system.

## 5. Conclusions

The presence of ASFV in domestic pigs in Lombardy has led to important economic and social repercussions for the entire pig sector. In both epidemics, the infection was rapidly eradicated in domestic pigs, but persisted in wild boars, and this represents a constant threat to domestic pigs.

Given the lack of vaccines and the resistance characteristics of ASFV in the environment, biosecurity is the only tool currently available to prevent and contain the spread of ASFV and must be implemented to interrupt the cycle of transmission of the infection from wild to domestic pigs, between pig farms, and also to prevent pigs from transmitting the infection back to wild boars.

Regarding biosecurity, in general it can be said that the level of application of biosecurity in the outbreaks was varied, better in the more industrial farms, less so in the smaller ones. An element of risk that emerged in all outbreaks is the location of the disinfection point, which in all outbreaks was placed inside the farm, even if outside the area strictly dedicated to pig breeding (the so-called clean area). This is permitted by current legislation but to be effective requires constant and correct application of cleaning and disinfection procedures of all vehicles or tools brought into the farm, including farm vehicles used for agricultural works, which in most cases were housed on the farm. In general, the mixing of different agricultural activities in the same facility makes it more difficult to effectively maintain farm biosecurity. An in-depth analysis of these data will be carried out soon.

## Figures and Tables

**Table 1:** ASF in Lombardy: attack rates in commercial farms in 2023 and 2024, by province and region.

Provinces _*(Year of Infection)*_	Number of Outbreaks *	Number of Commercial Farms **	Percentage of Outbreaks
PV ***_(2023)_***	9	181	5.0%
PV ***_(2024)_***	13	178	7.3%
MI ***_(2024)_***	2	85	2.4%
LO ***_(2024)_***	6	164	3.7%
Lombardy (all provinces 2023)	9	2416	0.4%
Lombardy(all provinces 2024)	21	2439	0.9%

* Data source: SIMAN; ** data source: BDR (last update in 2023: 18 August 2023; last update in 2024: 25 July2024).

## Data Availability

Data are contained within the article and Supplementary Materials.

## Supplementary Materials

The following supporting information can be downloaded at https://www.mdpi.com/article/10.3390/v17030327/s1; Table S1: Risk factors identified in ASF outbreaks in 2023 and 2024.
