# Supplementary material for: Epidemiological Characteristics of the African Swine Fever Genotype II Epidemic in Domestic Pigs in Lombardy (Northern Italy) in 2023 and 2024
Source: Viruses. 2025 Feb 26;17(3):327. doi: 10.3390/v17030327 (PMC11946006; doi:10.3390/v17030327)
Supplement: Supplementary file 1 [file viruses-17-00327-s001.zip › viruses-3458129-supplementary.pdf]

Table S1. Risk factors identified in ASF outbreaks in 2023 and 2024.

	Farm Code	Production Type**	Wild Boar Contact	Same Property	Supply Chain	Proximity	Introduction of Pigs from ASF Outbreak	Swill Feeding	Movement of Vehicles Carcasses/ Feed/ Pigs	Supply Chain Vet/ Technician	Manteinance Works	Total	Cases §	Prevalence	Kernel †	
															Value	Level
Year 2023																
1	095PVXXX	I	X									**1**	-	-	-	-
2	190PVXXX*	I										**0**	-	-	-	-
3	190PVXXX	I		X	X	X			X			**4**	-	-	-	-
4	190PVXXX	I		X	X	X			X			**4**	-	-	-	-
5	190PVXXX	I - F				X						**1**	-	-	-	-
6	190PVXXX	I				X						**1**	-	-	-	-
7	061PVXXX	I				X			X			**2**	-	-	-	-
8	151PVXXX	I			X				X			**2**	-	-	-	-
9	113PVXXX*	RCA			X					X		**2**	-	-	-	-
Year 2024																
1	022MIXXX	RCA	X									**1**	45	7.76%	0.014	2
2	102PVXXX	RCA			X						X	**2**	13	0.10%	0.014	2
3	068PVXXX	RCA			X							**1**	10	0.51%	0.015	2
4	236MIXXX	RCA										**0**	64	52.46%	0.015	2
5	160PVXXX	I				X			X	X		**3**	4	0.36%	0.059	4
6	139PVXXX	I			X					X		**2**	-	-	0.047	4
7	087PVXXX	I			X	X			X	X		**4**	2	0.10%	0.059	4
8	164PVXXX	I							X	X		**2**	3	0.01%	0.014	2
9	176PVXXX	RCA				X			X	X		**3**	1	0.03%	0.052	4
10	141PVXXX	I			X				X		X	**3**	4	0.21%	0.034	3
11	077PVXXX	I			X	X						**2**	1	0.12%	0.058	4
12	058PVXXX	RCA										**0**	10	0.74%	0.023	3
13	070PVXXX	RCA								X		**1**	8	0.16%	0.032	3
14	050LOXXX	I		X	X				X	X		**4**	22	3.26%	0.056	4
15	004LOXXX	RCA		X	X				X	X		**4**	6	0.25%	0.033	3
16	036LOXXX	I		X	X				X	X		**4**	12	0.81%	0.051	4
17	087PVXXX	I				X			X			**2**	1	0.07%	0.059	4
18	004PVXXX	I			X							**1**	4	0.05%	0.020	2
19	036LOXXX	RCA			X	X						**2**	9	0.18%	0.048	4
20	015LOXXX	I				X						**1**	72	5.39%	0.050	4
21	045LOXXX	RCA									X	**1**	16	0.25%	0.022	3
**TOTAL**			**2**	**5**	**15**	**12**	**0**	**0**	**13**	**10**	**3**					

* Outgoing movements of pigs. ** I = Fattening Farm; F = Family-run Farm; RCA= Open Cycle Breeding Farm. § Animals with clinical signs and/or lesions. † Kernel Level ('Natural Breaks' interpolation) 1 = 0.001-0.006; 2 = 0.007-0.02; 3 = 0.021- 0.039; 4 = 0.04-0.061.
